# Reactive carbonyl species function downstream of reactive oxygen species in chitosan‐induced stomatal closure

**DOI:** 10.1111/ppl.70094

**Published:** 2025-01-31

**Authors:** Israt Jahan, Md. Moshiul Islam, Toshiyuki Nakamura, Yoshimasa Nakamura, Shintaro Munemasa, Jun'ichi Mano, Yoshiyuki Murata

**Affiliations:** ^1^ Graduate School of Environmental and Life Science Okayama University Okayama Japan; ^2^ Department of Agronomy Bangabandhu Sheikh Mujibur Rahman Agricultural University Gazipur Bangladesh; ^3^ Science Research Center Yamaguchi University Yamaguchi Japan

## Abstract

An elicitor, chitosan (CHT), induces stomatal closure in plants, which is accompanied by salicylhydroxamic acid (SHAM)‐sensitive peroxidases‐mediated reactive oxygen species (ROS) production in guard cells. Reactive carbonyl species (RCS) function downstream of ROS in abscisic acid (ABA) and methyl jasmonate (MeJA) signalling in guard cells. However, the involvement of RCS in CHT‐induced stomatal closure is still unknown. In this study, we used transgenic tobacco (*Nicotiana tabacum*) plants overexpressing *Arabidopsis thaliana* 2‐alkenal reductase (AER‐OE tobacco) and Arabidopsis wild‐type (WT) plants to investigate whether RCS is involved in CHT‐induced stomatal closure. Chitosan‐induced stomatal closure was inhibited in the tobacco AER‐OE plants. In the WT tobacco and Arabidopsis plants, CHT‐induced stomatal closure was inhibited by RCS scavengers, carnosine and pyridoxamine. Chitosan significantly increased RCS production in the WT tobacco and Arabidopsis, but in the tobacco AER‐OE plants, chitosan did not increase significantly RCS accumulation. Moreover, neither the application of RCS scavengers to both WT plants nor scavenging RCS by AER‐OE affected the CHT‐induced ROS accumulation. However, treatment with a peroxidase inhibitor, SHAM, significantly inhibited CHT‐induced RCS accumulation in WT tobacco and Arabidopsis plants. Taken together, these results suggest that RCS acts downstream of ROS production in CHT signalling in guard cells of *A. thaliana* and *N. tabacum*.

## 1 INTRODUCTION

The epidermis of leaves has tiny pores called stomata, which are surrounded by pairs of guard cells. Guard cells regulate stomatal apertures in response to various biotic and abiotic stimuli and control gaseous fluxes and transpiration (Shimazaki et al., 2007; Murata et al., 2015). In response to various stresses, plants produce hormones like abscisic acid (ABA), methyl jasmonate (MeJA), and salicylic acid, which induce stomatal closure by modulating different signalling components (Lee, 1998; Schroeder et al., 2001; Mori et al., 2001; Suhita et al., 2004; Munemasa et al., 2007; Wasternack, 2007; Huang et al., 2008; Okuma et al., 2014) although it has been reported that jasmonic acid and salicylic acid play minor roles in guard‐cell signalling (Zamora et al., 2021). Stomatal closure is induced by plant hormones and elicitors (Klüsener et al., 2002). Chitosan (CHT), an elicitor, is a deacetylated derivative of chitin, which is found in fungal cell walls, arthropod exoskeletons, and crustacean shells. Chitosan enhances growth and yield performance and triggers plant defense responses (Doares et al., 1995, Gornik et al., 2008; De Vega et al., 2021; Chen et al., 2023; Ibrahim et al., 2023). Furthermore, CHT induces stomatal closure in *Solanum lycopersicum* and *Commelina communis* (Lee et al., 1999), *Arabidopsis thaliana* (Klüsener et al., 2002; Khokon et al., 2010; Salam et al., 2012; Jahan et al., 2024), and *Pisum sativum* (Srivastava et al., 2009).

In hormone and elicitor signalling in guard cells, reactive oxygen species (ROS) function as second messengers. The observation of intracellular ROS using 2′,7′‐dichlorodihydrofluorescein diacetate showed that ABA‐induced ROS accumulation was impaired by the *atrbohD atrbohF* double mutation and by diphenylene iodonium chloride (Suhita et al., 2004), which is an inhibitor of flavoproteins including NADPH oxidase, and that MeJA‐induced ROS accumulation was impaired by diphenylene iodonium chloride (Suhita et al., 2004). Moreover, the observation of extracellular ROS using 3,3’‐Diaminobenzidine showed that salicylic acid‐induced (Khokon et al., 2011) and CHT‐induced ROS accumulation was disrupted by salicylhydroxamic acid (SHAM) (Supporting information, Figure S2), which is not only an inhibitor of extracellular peroxidases but also an inhibitor of alternative oxidases in mitochondria (Dupont & Rustin, 1987; Mori et al., 2001). Hence, it is considered that ABA and MeJA induced ROS production by plasma membrane NAD(P)H oxidases (Pei et al., 2000; Kwak et al., 2003; Munemasa et al., 2007) while salicylic acid and CHT induced ROS production by SHAM‐sensitive peroxidases (Mori et al., 2001; Khokon et al., 2010, 2011; Salam et al., 2012).

Reactive oxygen species oxidize membrane lipids to form lipid peroxides. Decomposition of lipid peroxides via enzymatic and non‐enzymatic radical‐catalyzed reactions generates reactive compounds, including aldehydes, ketones and hydroxy acids (Blée, 1998; Mueller, 2004; Mosblech et al., 2009; Mano, 2012). Aldehydes and ketones comprising *α,β*‐unsaturated bonds are termed reactive carbonyl species (RCS) because of their high electrophilicity and reactivity (Mano, 2012). Reactive carbonyl species mediate stomatal closure induced by ABA and MeJA in *A. thaliana* (Islam et al., 2019, 2020; Rhaman et al., 2020) and in tobacco (*Nicotiana tabacum*) (Islam et al., 2016, 2020). However, the role of RCS in CHT‐induced stomatal closure is still unclear.

Abscisic acid‐ and MeJA‐induced stomatal closure was inhibited in the guard cells of transgenic tobacco plants overexpressing 2‐alkenal reductase (AER‐OE plants) (Islam et al., 2016, 2020), where AER catalyzes a RCS scavenging reaction. The AER overexpression and application of RCS scavengers, carnosine and pyridoxamine, inhibited ABA‐ and MeJA‐induced RCS production but did not impair the ABA‐ and MeJA‐induced ROS accumulation in the guard cells (Islam et al., 2016, 2019, 2020) although the RCS scavengers can affect ROS accumulation in some instances (Boldyrev et al., 2004; Wang et al., 2015). Therefore, it is evident that RCS acts as signal molecules downstream of ROS in ABA and MeJA signalling in guard cells. However, how the generated ROS is transduced into downstream signalling components in CHT signalling in guard cells remains to be clarified.

Therefore, to investigate the involvement of RCS in guard cell CHT signalling, we examined stomatal movements, ROS production, and RCS accumulation using tobacco WT and AER‐OE plants, and Arabidopsis WT plants.

## 2 MATERIALS AND METHODS

### 2.1 Plant materials and growth conditions

Arabidopsis *(A. thaliana)* WT (Columbia‐0), tobacco (*N. tabacum*) WT, and three transgenic tobacco lines overexpressing 2‐alkenal reductase (At5g16970), P1#11, P1#14, and P1#18 (AER‐OE plants; Mano et al., 2005) were used. These plants were grown as described previously (Jahan et al., 2024).

### 2.2 Measurement of stomatal aperture

Stomatal apertures were measured as described previously (Islam et al., 2016). Excised leaves were floated on stomatal assay buffer containing 5 mM KCl, 50 μM CaCl_2_, and 10 mM 2‐(*N*‐morpholino)ethanesulfonic acid‐Tris (pH 5.6) for 2 h in the light. Chitosan was partially purified from commercial chitosan (CAT no. 448869; Sigma Aldrich), according to Hadwiger and Beckman (1980), and was added and incubated for 2 h in the light. The RCS scavengers were added 30 min before chitosan application. In each individual experiment, the widths of twenty stomatal apertures were measured and considered as one set. The experiment was repeated three times under the same condition to get three sets of stomata. Then, the mean from the averages of three sets (total 60 stomata) was calculated, and the data was presented in each bar.

### 2.3 Measurement of ROS production in guard cells

Production of ROS in guard cells of Arabidopsis and tobacco was examined using 2′,7′‐dichlorodihydrofluorescein diacetate (Sigma) as described previously (Islam et al., 2016). Epidermal tissues were incubated for 3 h in the stomatal assay solution containing 5 mM KCl, 50 μM CaCl_2_, and 10 mM 2‐(*N*‐morpholino)ethanesulfonic acid‐Tris (pH 5.6). Then, 50 μM 2′,7′‐dichlorodihydrofluorescein diacetate was added to the solution and incubated for 30 min under dark conditions. The dye‐loaded epidermal tissues were then treated with 50 μg/mL CHT for 20 min. Fluorescence images of guard cells were captured under a fluorescence microscope (Biozero BZ‐8000, KEYENCE) and the fluorescence intensities of guard cells were analyzed using ImageJ 1.42q software (National Institutes of Health).

### 2.4 Identification and quantification of carbonyls

Epidermal tissues were floated on stomatal assay solution containing 5 mM KCl, 50 μM CaCl_2_, and 10 mM 2‐(*N*‐morpholino)ethanesulfonic acid‐Tris (pH 5.6) for 2 h in the light. Then, 50 μg mL^−1^ of CHT was added and kept in the light for 30 min. Carbonyls were extracted and derivatized from the epidermal tissues with 2,4‐dinitrophenylhydrazine. The dinitrophenylhydrazone‐carbonyl derivatives were then determined by reverse‐phase high‐performance liquid chromatography as described previously (Islam et al., 2016).

### 2.5 Statistical analysis

For data analysis, Student's *t*‐test and Tukey's test with one‐way ANOVA were performed to assess differences between mean values. A *P*‐value <0.05 was considered as statistically significant.

## 3 RESULTS

### 3.1 Reactive carbonyl species scavengers and overexpression of AER inhibited CHT‐induced stomatal closure in guard cells

**FIGURE 1:**
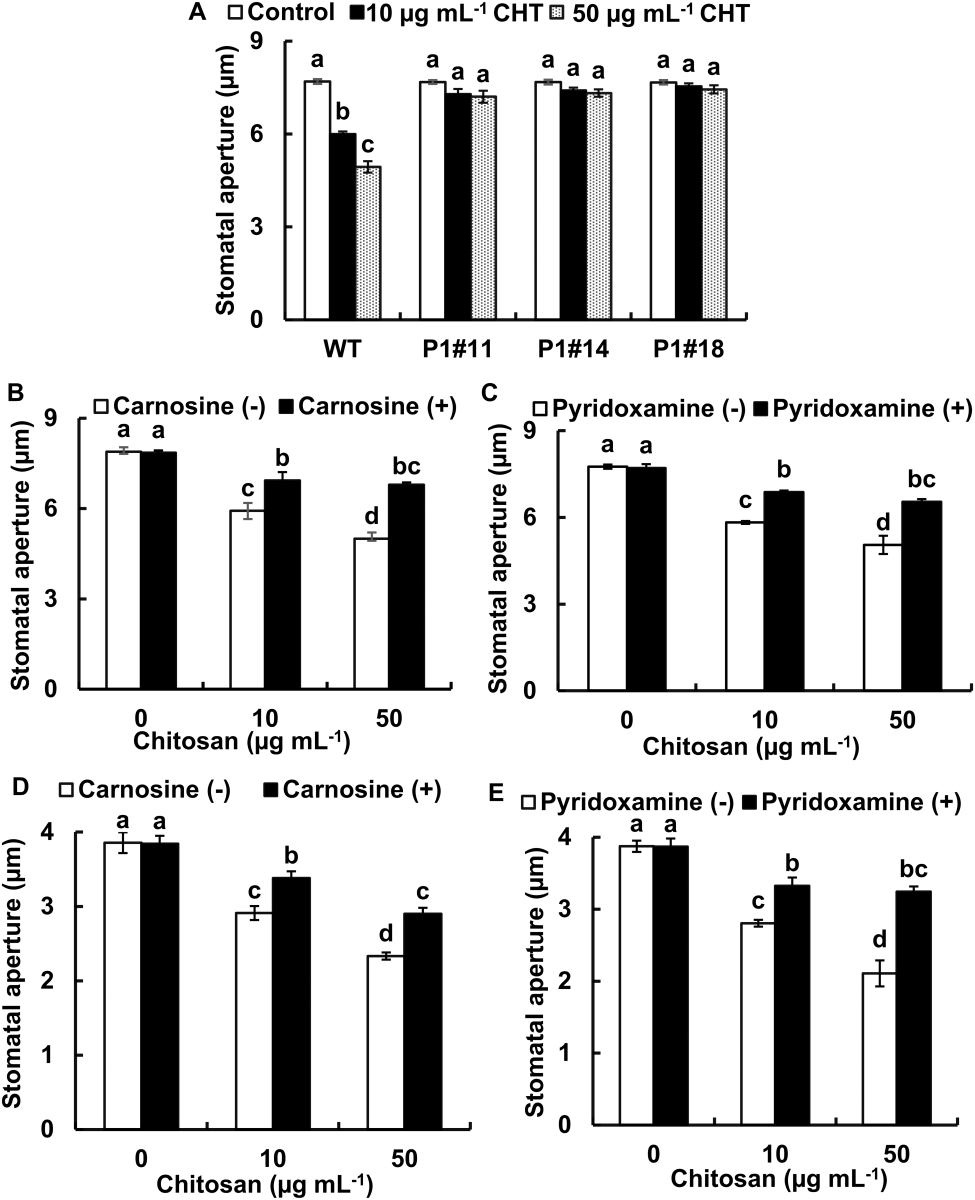
Chitosan‐induced stomatal closure in tobacco and Arabidopsis. Chitosan‐induced stomatal closure in the absence (A) or presence of RCS scavengers (B, D: 1 mM carnosine; C, E: 0.5 mM pyridoxamine) were examined using tobacco (A, B, C), WT and AER‐OE lines (P1#11, P1#14, P1#18), and using Arabidopsis (D, E). The RCS scavengers were added 30 min before CHT application. Each bar represents the averages from three independent experiments (total 60 stomata). Error bars indicate the standard error of the mean. Different letters represent significant differences at *p* < 0.05 by Tukey's test.

To determine the involvement of RCS in CHT‐induced stomatal closure, we measured stomatal apertures of tobacco AER‐OE plants in response to CHT (Figure 1). Chitosan at 10 and 50 μg mL^−1^ significantly induced stomatal closure in WT tobacco plants. Application of 10 and 50 μg mL^−1^ CHT did not significantly induce stomatal closure in tobacco AER‐OE plants, P1#11, P1#14, and P1#18, (Figure 1A).

To further investigate the role of RCS in CHT‐induced stomatal closure, we analyzed the effects of RCS scavengers on the stomatal closure in WT tobacco and Arabidopsis plants (Figure 1). Pretreatments with 1 mM carnosine and 0.5 mM pyridoxamine significantly inhibited stomatal closure induced by 10 and 50 μg mL^−1^ of CHT in WT tobacco (Figure 1B and C) and Arabidopsis (Figure 1D and E).

These results indicate that RCS is responsible for CHT‐induced stomatal closure in Arabidopsis and tobacco.

### 3.2 Chitosan induced RCS production in Arabidopsis and tobacco WT plants but not in tobacco AER‐OE plants

**FIGURE 2:**
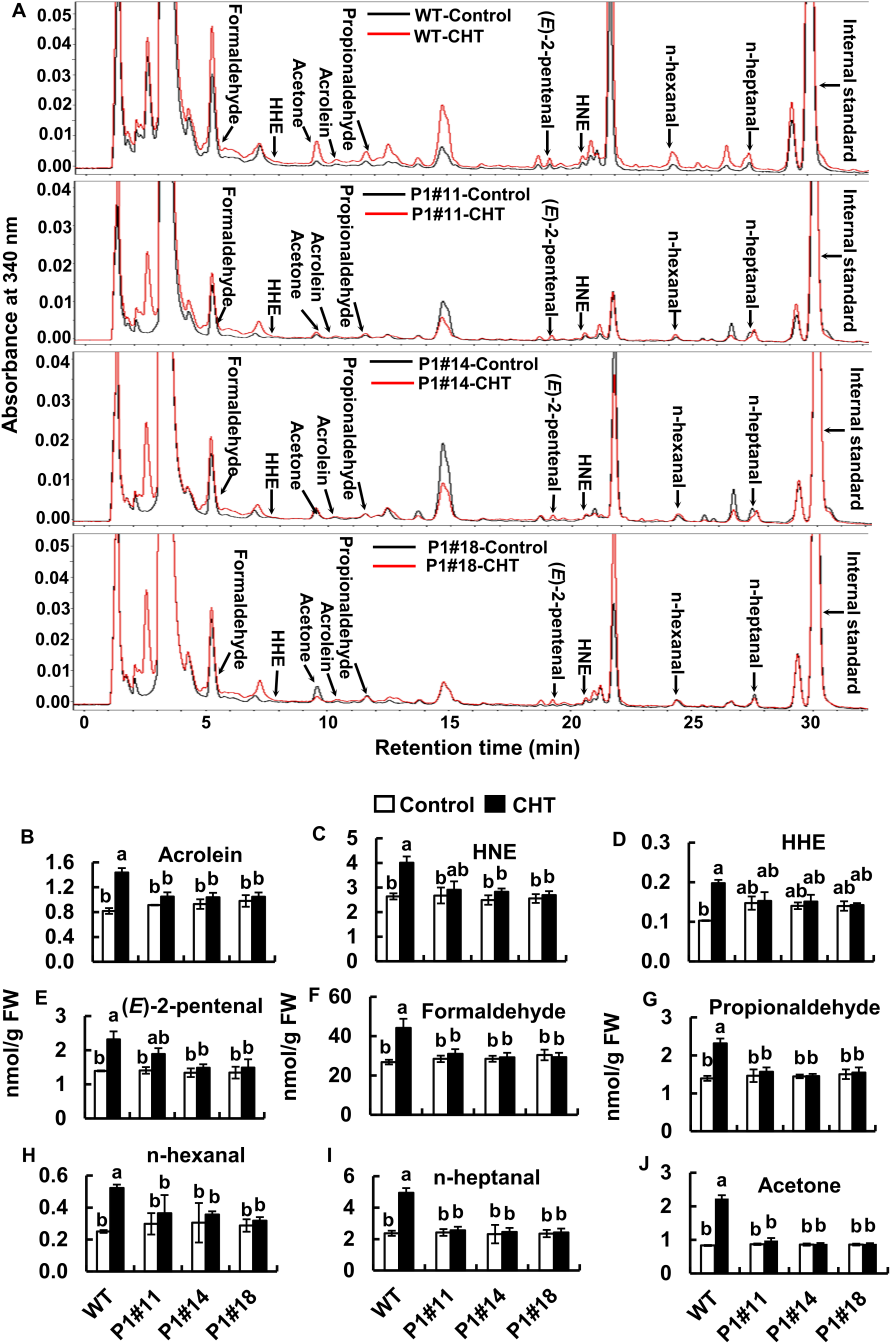
Effect of CHT on accumulation of aldehydes including RCS, in the epidermal tissues of tobacco, WT and AER‐OE lines (P1#11, P1#14, P1#18). (A) Representative chromatograms of the dinitrophenylhydrazone‐labeled carbonyls from epidermal tissues of tobacco treated with or without CHT are shown. Dinitrophenylhydrazone derivatives of carbonyls were detected at 340 nm. Carbonyls are labeled at the top of each peak. (B‐J) Contents of carbonyls in the epidermal tissues of tobacco with or without CHT treatment are shown. Epidermal tissues were incubated in the light for 2 h and then treated with CHT for 30 min. Error bars indicate the standard error of the mean. Different letters represent significant differences at *p* < 0.05 by Tukey's test. Note that HHE and HNE stand for 4‐hydroxy‐(*E*)‐2‐hexenal and 4‐hydroxy‐(*E*)‐2‐nonenal, respectively.

To elucidate the participation of RCS in CHT‐induced stomatal closure, we quantified RCS in tobacco WT and AER‐OE plants and Arabidopsis WT plants when treating these plants with CHT (Figures 2 and 4). Typical chromatograms of the tobacco WT and AER‐OE plants treated with or without CHT are presented in Figure 2A. In tobacco WT plants, the application of CHT at 50 μg/mL significantly increased the levels of four RCS and five non‐RCS carbonyls but not in tobacco AER‐OE plants (Figure 2B‐J). The increased RCS were acrolein, 4‐hydroxy‐(*E*)‐2‐nonenal, 4‐hydroxy‐(*E*)‐2‐hexenal, and (*E*)‐2‐pentenal (Figure 2B‐E). In CHT‐treated guard cells, the level of acrolein was increased by 1.76‐fold and 4‐hydroxy‐(*E*)‐2‐nonenal by 1.52‐fold (Figure 2B and C). The other increased non‐RCS carbonyls were formaldehyde, propionaldehyde, n‐hexanal, n‐heptanal, and acetone (Figure 2F‐J).

In Arabidopsis WT plants, the contents of RCS were significantly increased when epidermal tissues were treated with 50 μg/mL CHT (Figure 4). Typical chromatograms for the epidermal tissues treated with CHT or without CHT in Arabidopsis WT plants are shown in Figure 4A. The increased RCS were acrolein, 4‐hydroxy‐(*E*)‐2‐nonenal, 4‐hydroxy‐(*E*)‐2‐hexenal, (*E*)‐2‐pentenal, malondialdehyde, and crotonaldehyde (Figure 4C‐H). In CHT‐treated guard cells, the levels of acrolein were increased by 1.81‐fold and 4‐hydroxy‐(*E*)‐2‐nonenal by 1.52‐fold (Figure 4C and D). Non‐RCS carbonyls that also increased were formaldehyde, acetaldehyde, propionaldehyde, n‐hexanal, and n‐heptanal (Figure 4I‐M). These findings suggest that CHT‐induced stomatal closure requires RCS production in guard cells.

### 3.3 Reactive carbonyl species scavengers and overexpression of AER did not affect CHT‐induced ROS production

**FIGURE 3:**
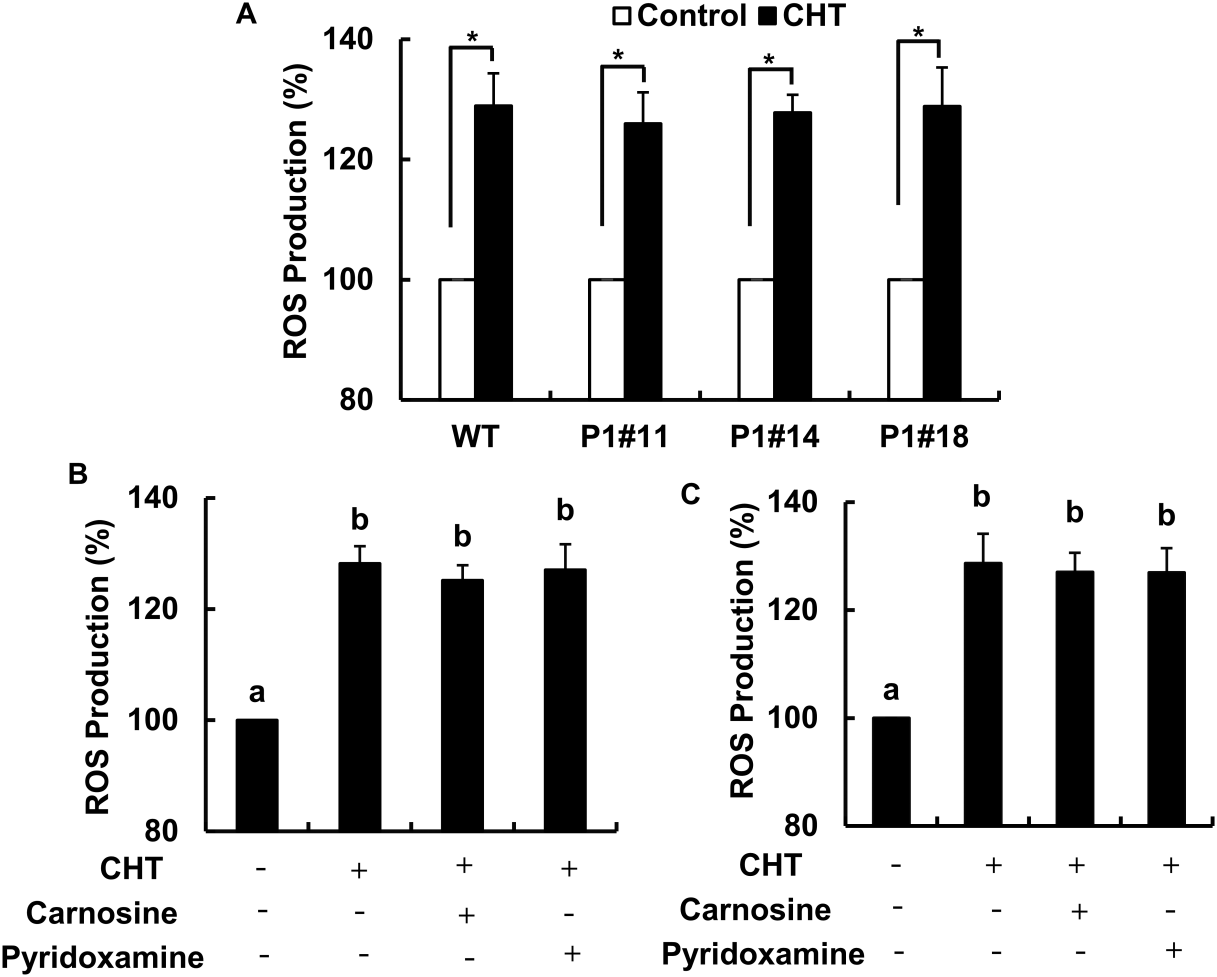
Chitosan‐induced ROS production in tobacco and Arabidopsis. Chitosan‐induced ROS production in guard cells was measured using tobacco (A: WT and AER‐OE lines (P1#11, P1#14, P1#18); B: WT) and Arabidopsis (C) treated with RCS scavengers (B, C: 1 mM carnosine and 0.5 mM pyridoxamine). The vertical scale represents the percentage of ROS production. Each bar represents the averages from three independent experiments (60 guard cells per bar). The fluorescent intensities were normalized to the control value considered at 100%. Error bars indicate the standard error of the mean. Statistical differences were analyzed using Student's *t*‐test. *, *p* < 0.05 (A). Different letters represent significant differences at *p* < 0.05 by Tukey's test (B, C).

We measured CHT‐induced ROS accumulation in tobacco WT and AER‐OE plants. Chitosan significantly induced ROS accumulation in the guard cells of WT plants (Figure 3). Overexpression of AER did not impair CHT‐induced ROS accumulation in three tobacco AER‐OE plants (Figure 3A). This result indicates that RCS is produced downstream of ROS generation in CHT‐induced stomatal closure.

We measured the effects of RCS scavengers on ROS levels in CHT‐treated Arabidopsis and tobacco WT plants to clarify that RCS is produced downstream of ROS (Figure 3). Treatment with 1 mM carnosine and 0.5 mM pyridoxamine did not impair CHT‐induced ROS production in the WT tobacco (Figure 3B) and Arabidopsis (Figure 3C).

**FIGURE 4:**
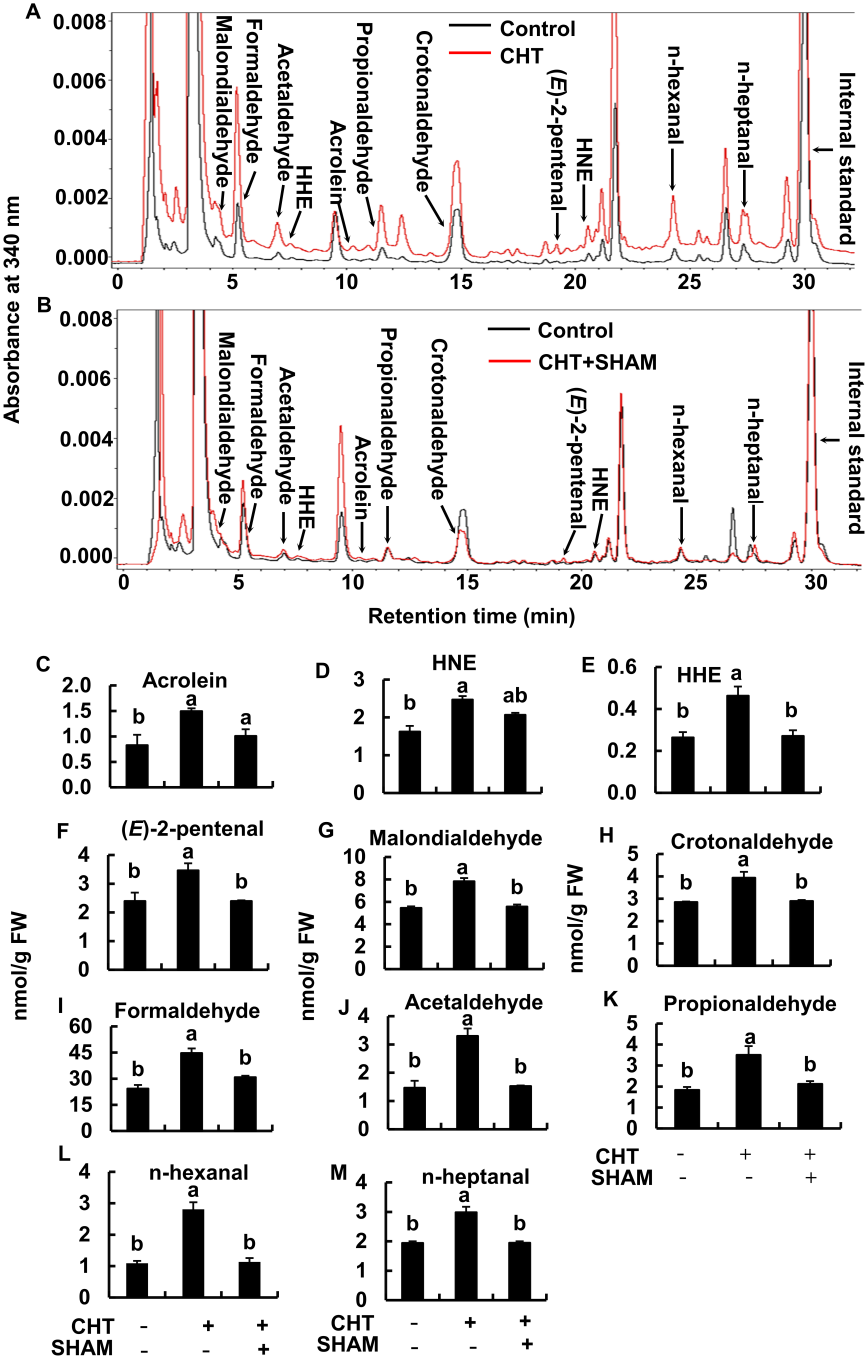
Effects of 2 mM SHAM on CHT‐induced accumulation of aldehydes including RCS in the epidermal tissues of Arabidopsis WT. Typical chromatograms of the dinitrophenylhydrazone‐carbonyl derivatives extracted from epidermal tissues of Arabidopsis treated with and without CHT in the absence (A) or presence (B) of 2 mM SHAM are shown. Dinitrophenylhydrazone derivatives of carbonyls were detected at 340 nm. Carbonyls are labeled at the top of each peak. (C‐M) Contents of carbonyls in the epidermal tissues of Arabidopsis WT plants. Epidermal tissues were incubated in the light for 2 h and then treated with CHT for 30 min. Two mM SHAM was applied 30 min prior to CHT application. Error bars indicate the standard error of the mean. Different letters represent significant differences at *p* < 0.05 by Tukey's test. Note that HHE and HNE stand for 4‐hydroxy‐(*E*)‐2‐hexenal and 4‐hydroxy‐(*E*)‐2‐nonenal, respectively.

**FIGURE 5:**
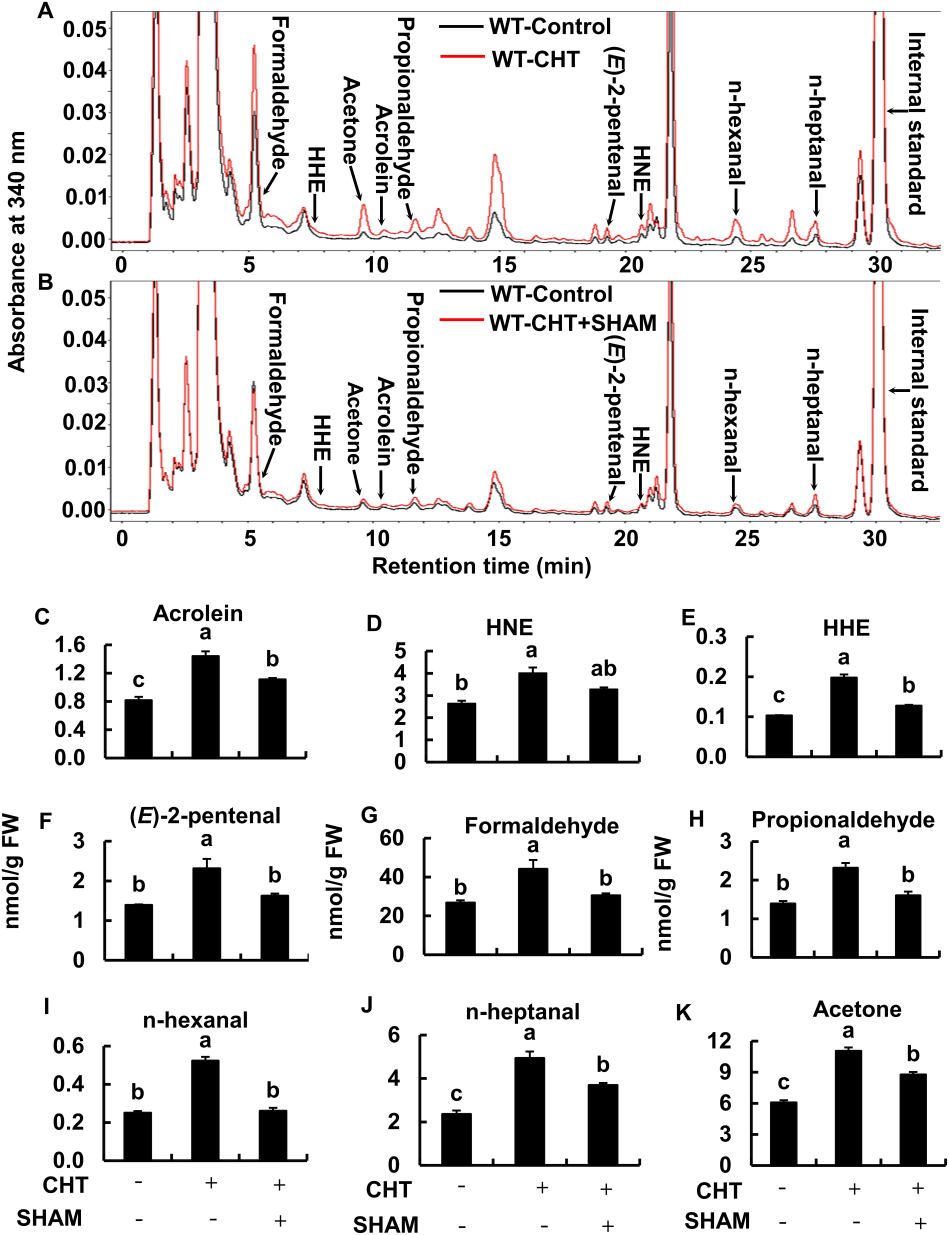
Effects of 2 mM SHAM on CHT‐induced accumulation of aldehydes including RCS in the epidermal tissues of tobacco WT. Typical chromatograms of the dinitrophenylhydrazone derivatives of carbonyls extracted from epidermal tissues of tobacco treated with and without CHT in the absence (A) or presence (B) of 2 mM SHAM are shown. Dinitrophenylhydrazone derivatives of carbonyls were detected at 340 nm. Carbonyls are labeled at the top of each peak. (C‐K) Contents of carbonyls in the epidermal tissues of tobacco. Epidermal tissues were incubated in the light for 2 h and then treated with CHT for 30 min. Two mM SHAM was applied 30 min prior to CHT application. Error bars indicate the standard error of the mean. Different letters represent significant differences at *p* < 0.05 by Tukey's test. The chromatograms and bars for control and CHT are the same as shown in Figure 2 for WT tobacco. Note that HHE and HNE stand for 4‐hydroxy‐(*E*)‐2‐hexenal and 4‐hydroxy‐(*E*)‐2‐nonenal, respectively.

To further confirm that RCS generation depends on ROS production in guard cells, we examined the effect of a peroxidase inhibitor, salicylhydroxamic acid (SHAM), on the RCS accumulation in the CHT‐treated epidermal tissues of Arabidopsis and tobacco WT plants (Figure 4 and 5). Typical chromatograms for the WT Arabidopsis and tobacco plants treated with 50 μg mL^−1^ of CHT with or without 2 mM SHAM are presented in Figure 4A and B, and Figure 5A and B, respectively. The application of 2 mM SHAM significantly inhibited the CHT‐induced accumulation of aldehydes, including RCS in WT Arabidopsis (Figure 4C‐M) and tobacco (Figure 5C‐K). Taken together, these results suggest that RCS acts as a signal mediator downstream of ROS accumulation in CHT‐induced stomatal closure.

## 4 DISCUSSION

This study elucidated the involvement of reactive carbonyl species in stomatal closure induced by chitosan (CHT) in guard cells. Previous studies found that CHT induces stomatal closure along with salicylhydroxamic acid (SHAM)‐sensitive peroxidases‐mediating ROS production in several plant species. However, how RCS functions in ROS signalling in CHT‐induced stomatal closure is still unclear.

When ROS oxidize the membrane lipids, they produce lipid peroxides. Redox catalysts decompose lipid peroxides to form various aldehydes and ketones (i.e., oxylipin carbonyls; Farmer and Mueller, 2013). Among the oxylipin carbonyls, the *α,β*‐unsaturated carbonyls are termed RCS, which have high reactivity and cytotoxicity (Esterbauer et al., 1991; Alméras et al., 2003). In plants, RCS and several saturated oxylipin carbonyls (non‐RCS) are formed constitutively in leaves and roots, and their levels are increased under various stresses (Mano et al., 2010). Although RCS and these non‐RCS carbonyls have different reactivities, they share common properties as electrophiles (Schauenstein et al., 1977). In plants, several RCS and non‐RCS oxylipin carbonyls were involved in the programmed cell death process in oxidatively stressed cells (Biswas & Mano, 2015). The contents of several RCS and non‐RCS were increased in the guard‐cell signalling pathway involved in ABA and MeJA (Islam et al., 2016, 2019, 2020).

Previous studies using RCS scavengers and tobacco AER‐OE plants revealed that both stomatal closure and RCS accumulation induced by ABA and MeJA were impaired in tobacco AER‐OE plants and in tobacco and Arabidopsis WT plants with the application of scavengers (Islam et al., 2016, 2019, 2020). In this study, chitosan elicited stomatal closure and accumulation of several kinds of RCS and non‐RCS carbonyls in WT tobacco (Figures 1 and 2) and Arabidopsis (Figures 1 and 4) but not in the tobacco AER‐OE plants (Figures 1 and 2). The AER could scavenge the longer chain precursors of aldehydes containing the *α*,*β*‐unsaturated carbonyl structures and thus suppressing the generation of non‐RCS carbonyls (Sultana et al., 2024). Moreover, this study shows that CHT failed to induce stomatal closure in the presence of the RCS scavengers in WT tobacco (Figure 1B and C) and Arabidopsis (Figure 1D and E). These results suggest that RCS plays as an essential factor in CHT‐induced stomatal closure in Arabidopsis and tobacco.

Chitosan and salicylic acid induced ROS production by SHAM‐sensitive peroxidases (Mori et al., 2001; Khokon et al., 2010, 2011; Salam et al., 2012) while ABA and MeJA induced NAD(P)H oxidases‐catalyzing ROS production (Pei et al., 2000; Kwak et al., 2003; Munemasa et al., 2007). Abscisic acid‐ and MeJA‐induced ROS production was not impaired in tobacco AER‐OE plants (Islam et al., 2016, 2020) and by the application of RCS scavengers in WT tobacco and Arabidopsis plants (Islam et al., 2019, 2020). In this study, CHT‐induced ROS production was observed in tobacco AER‐OE plants (Figure 3A) and with the application of RCS scavengers in WT tobacco (Figure 3B) and Arabidopsis plants (Figure 3C). We found different ROS levels in the tobacco WT and AER‐OE lines in the absence of CHT (Supporting Information, Figure S1). However, the difference is not likely to affect stomatal apertures because increased constitutive ROS accumulation did not significantly affect the stomatal aperture (Jannat et al., 2011). In WT tobacco, ABA‐induced RCS production was impaired when guard‐cell NAD(P)H oxidases were inhibited by diphenylene iodonium chloride (Islam et al., 2016). In this study, suppression of CHT‐induced ROS production by a peroxidase inhibitor, SHAM, significantly impaired CHT‐induced RCS accumulation in Arabidopsis (Figure 4C‐H) and tobacco WT plants (Figure 5C‐K). These results suggest that RCS is produced downstream of ROS in the CHT‐signaling pathway in guard cells.

Chitosan induces stomatal closure along with depletion of intracellular GSH in guard cells (Jahan et al., 2024). As GSH is a nucleophile, its decrease can be attributed to enzymatical or non‐enzymatical conjugation with electrophiles. Reactive carbonyl species can react with GSH to form conjugates (Esterbauer et al., 1975; Davoine et al., 2006). Hence, it can be speculated that CHT signalling is regulated by RCS via the conjugate formation in guard cells.

## AUTHOR CONTRIBUTIONS

I.J., S.M., and Y.M. designed the research. I.J. performed all experiments with support of M.M.I., S.M., and T.N. I.J. analyzed data with support of M.M.I. T.N. and Y.N. provided suggestions. I.J. and Y.M. wrote the manuscript.

## FUNDING INFORMATION

This work was supported by the Japan Society for the Promotion of Science (JSPS) KAKENHI [Grant Number 22H02303 (to Y.M., S.M., and T.N.)] and Japan Society for the Promotion of Science Bilateral Collaborations [Grant Number JPJSBP120219925 (to I.J., Y.M., S.M., and T.N.)].

## CONFLICT OF INTEREST STATEMENT

No potential conflict of interest was reported by the authors.

## Supporting information


**Data S1.** Supporting Information.

## Data Availability

The analyzed data sets generated during the study are available from the corresponding author upon reasonable request.
