# Supplementary material for: Reactive carbonyl species function downstream of reactive oxygen species in chitosan‐induced stomatal closure
Source: Physiol Plant. 2025 Jan 31;177(1):e70094. doi: 10.1111/ppl.70094 (PMC11783587; doi:10.1111/ppl.70094)
Supplement: Supplementary file 1 — Data S1. Supporting Information. [file PPL-177-e70094-s001.pdf]

SUPPLEMENTARY MATERIAL

# Reactive carbonyl species function downstream of reactive oxygen species in chitosan-induced stomatal closure

**Israt Jahan[1], Md. Moshiul Islam[1,2], Toshiyuki Nakamura[1], Yoshimasa Nakamura[1], Shintaro Munemasa[1], Jun'ichi Mano[3], and Yoshiyuki Murata[1]***

[1] Graduate School of Environmental and Life Science, Okayama University, Okayama 700-8530, Japan

[2] Department of Agronomy, Bangabandhu Sheikh Mujibur Rahman Agricultural University, Gazipur 1706, Bangladesh

[3] Science Research Center, Yamaguchi University, Yoshida 1677-1, Yamaguchi 753-8515, Japan

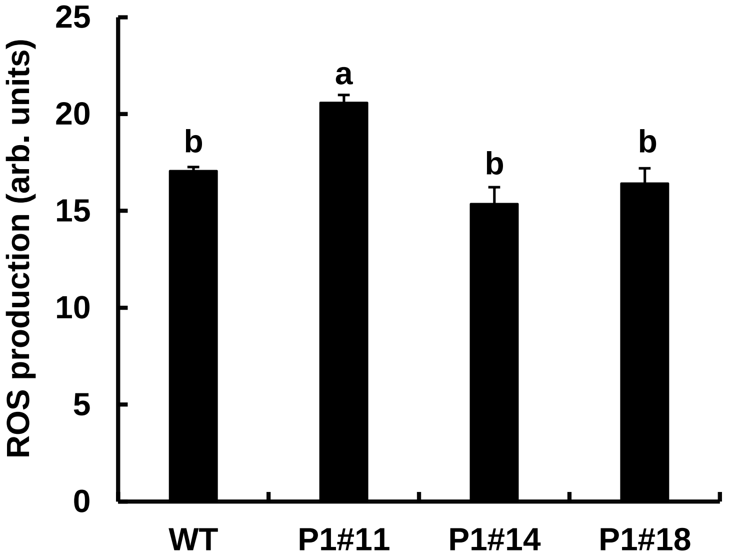


**Supporting information Figure S1:** Reactive oxygen species production in guard cells of tobacco WT and AER-OE lines (P1#11, P1#14, P1#18) in absence of CHT treatment. The vertical scale represents the fluorescence intensities. Each bar represents the averages from three independent experiments (60 guard cells per bar). Error bars indicate the standard error of the mean. Different letters represent significant differences at $P < 0.05$ by Tukey’s test.

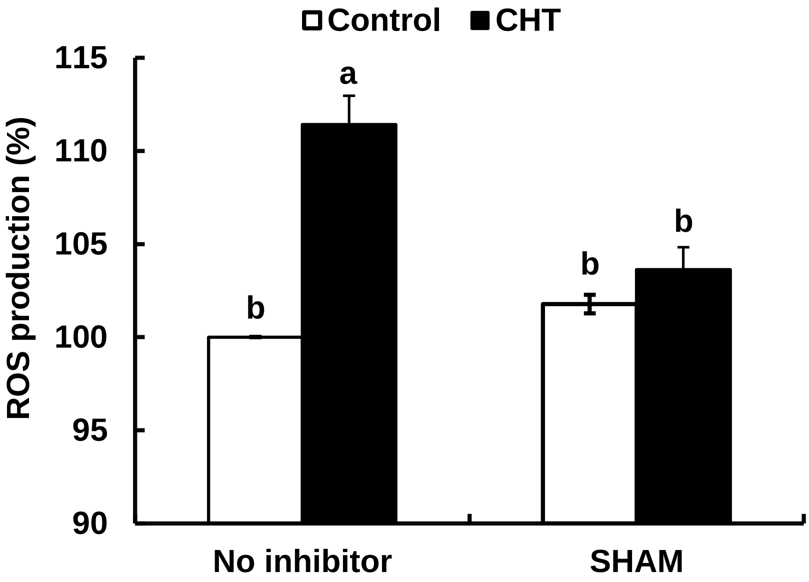


**Supporting information Figure S2:** The effect of 2 mM salicylhydroxamic acid (SHAM) on CHT-induced apoplastic ROS production in whole leaves of Arabidopsis WT plants using the ROS-sensitive dye, 3,3'-Diaminobenzidine (DAB). Detached rosette leaves of Arabidopsis plants were vacuum infiltrated with DAB assay buffer containing 1 mg mg $mL^{-1}$ DAB, 5 mM KCl, 50 μM $CaCl_2$, and 10 mM 2-(*N*-morpholino)ethanesulfonic acid-Tris (pH 5.6). Infiltrated leaves were incubated in DAB assay buffer supplemented with 0.1% Tween 20 for 2 h in the light. Then, chemical was added and the leaves were incubated in the light for 2 h. The leaves were treated with SHAM, for 30 min before chemical application. Pixel intensities of oxidized DAB were normalized to the control value taken as 100. Averages from three independent experiments (12 leaves per bar in total) are shown. Error bars indicate the standard error of the mean. Different letters represent significant differences at $P < 0.05$ by Tukey's test.
